# Editorial Comment: Role of Diffusion-Weighted Magnetic Resonance Imaging (DWMRI) in Assessment of Primary Penile Tumor Characteristics and Its Correlations With Inguinal Lymph Node Metastasis: A Prospective Study

**DOI:** 10.1590/S1677-5538.IBJU.2020.06.05

**Published:** 2020-09-02

**Authors:** Gustavo Cardoso Guimarães

**Affiliations:** 1 Departamento de Oncologia Cirúrgica Beneficência Portuguesa de São Paulo São PauloSP Brasil Chefe do Departamento de Oncologia Cirúrgica, Beneficência Portuguesa de São Paulo, São Paulo, SP, Brasil

Sasanka Kumar Barua ^1^, Pranab K Kaman ^1^, Saumar Jyoti Baruah ^1^, Rajeev T P ^1^, Puskal K Bagchi ^1^, Debanga Sarma ^1^, Yashasvi Singh ^1^

*^1^ Department of Urology, Gauhati Medical College Hospital, Guwahati, Assam, India.*

World J Oncol. 2018;9(5-6):145-150.

**DOI: 10.14740/wjon1138w | ACCESS: 10.14740/wjon1138w**

## COMMENT

In this interesting paper, Dr Barua and colleagues at Gauhati Medical College Hospital, India, prospectively investigated the role of Diffusion-Weighted Magnetic Resonance in assessing the characteristics of the primary penile tumor and its correlation with the presence or absence of lymph node metastases.

Lymph node metastasis in penile cancer is one of the most important prognostic factors ( 1 ). And finding ways to predict the risk of developing metastasis may be crucial for a better therapeutic approach and selecting candidates for lymphadenectomy.

In this study they evaluated 26 patients with penile tumors with Diffusion-Weighted Magnetic Resonance Imaging (DWMRI) and the apparent diffusion coefficient (ADC) values of primary tumor were compared with histological characteristics after surgical lymph node dissection. The inclusion criteria comprised of all carcinoma penis with non-palpable inguinal LN and normal sized LN on imaging.

The mean ADC values for grade 1, grade 2 and grade 3 were 0.89 × 10-3, 0.82 × 10-3 and 0.80 × 10-3 mm2/s, respectively. The ADC value of < 0.95 × 10-3 mm2/s was positively correlated with pathological LN presence within normal sized LN. With mean ADC value of 0.87 × 10-3 ± 0.11 × 10-3 mm2/s, sensitivity and positive predictive values for primary penile cancer were 100% and 84.61%, respectively. The sensitivity and specificity of predicting LN metastasis by DWMRI were 87.22% and 80.90%, respectively.

These interesting findings raise the possibility that DWMRI may prove to be a useful test in selecting patients not to undergo lymphadenectomy in the future. However, studies with larger series are necessary to prove this hypothesis.
